# Controls of Sediment Nitrogen Dynamics in Tropical Coastal Lagoons

**DOI:** 10.1371/journal.pone.0155586

**Published:** 2016-05-13

**Authors:** Alex Enrich-Prast, Viviane Figueiredo, Francisco de Assis Esteves, Lars Peter Nielsen

**Affiliations:** 1 Laboratório de Biogeoquímica, Departamento de Ecologia, Instituto de Biologia, Universidade Federal do Rio de Janeiro, Rio de Janeiro, Rio de Janeiro, Brazil; 2 Department of Environmental Change, Linköping University, Linköping, Sweden; 3 Departamento de Geoquímica, Universidade Federal Fluminense, Niterói, Rio de Janeiro, Brazil; 4 Laboratório de Limnologia, Departamento de Ecologia, Instituto de Biologia, Universidade Federal do Rio de Janeiro, Rio de Janeiro, Rio de Janeiro, Brazil; 5 Núcleo de Pesquisas em Ecologia e Desenvolvimento Sócio-ambiental de Macaé, Universidade Federal do Rio de Janeiro, Macaé, Rio de Janeiro, Brazil; 6 Department of Biology, University of Aarhus, Aarhus, Denmark; CAS, CHINA

## Abstract

Sediment denitrification rates seem to be lower in tropical environments than in temperate environments. Using the isotope pairing technique, we measured actual denitrification rates in the sediment of tropical coastal lagoons. To explain the low denitrification rates observed at all study sites (<5 μmol N_2_ m^-2^ h^-1^), we also evaluated potential oxygen (O_2_) consumption, potential nitrification, potential denitrification, potential anammox, and estimated dissimilatory nitrate (NO_3_^-^) reduction to ammonium (NH_4_^+^; DNRA) in the sediment. ^15^NO_3_^-^ and ^15^NH_4_^+^ conversion was measured in oxic and anoxic slurries from the sediment surface. Sediment potential O_2_ consumption was used as a proxy for overall mineralization activity. Actual denitrification rates and different potential nitrogen (N) oxidation and reduction processes were significantly correlated with potential O_2_ consumption. The contribution of potential nitrification to total O_2_ consumption decreased from contributing 9% at sites with the lowest sediment mineralization rates to less than 0.1% at sites with the highest rates. NO_3_^-^ reduction switched completely from potential denitrification to estimated DNRA. Ammonium oxidation and nitrite (NO_2_^-^) reduction by potential anammox contributed up to 3% in sediments with the lowest sediment mineralization rates. The majority of these patterns could be explained by variations in the microbial environments from stable and largely oxic conditions at low sediment mineralization sites to more variable conditions and the prevalences of anaerobic microorganisms at high sediment mineralization sites. Furthermore, the presence of algal and microbial mats on the sediment had a significant effect on all studied processes. We propose a theoretical model based on low and high sediment mineralization rates to explain the growth, activity, and distribution of microorganisms carrying out denitrification and DNRA in sediments that can explain the dominance or coexistence of DNRA and denitrification processes. The results presented here show that the potential activity of anaerobic nitrate-reducing organisms is not dependent on the availability of environmental NO_3_^-^.

## Introduction

Denitrification rates have been predominantly measured in temperate regions with ranges varying by orders of magnitude, whereas tropical environments have been underrepresented [1–2]. The few studies performed to date in aquatic tropical environments have reported relatively low or absent denitrification rates in sediments obtained from coastal lagoons [3, 4], mangroves [5], floodplain lakes from Pantanal [6] and Amazon [7–10] regions, and streams [2]. In most of these environments, the nitrate (NO_**3**_^**-**^) concentration in the water column was also relatively low.Oxygen (O_2_) in the sediment is a determining factor in nitrogen (N) transformation [11]. The mineralization of organic matter, a fundamental process that supports the N cycle (as well as other cycles) by providing substrates such inorganic N (ammonium: NH_4_^+^ and NO_3_^-^), is strongly positively correlated with oxygen consumption because O_2_ is consumed during both aerobic respiration and oxidation of anaerobic metabolism products [11].

Two anaerobic biological processes, denitrification and anammox, convert inorganic N into atmospheric dinitrogen (N_2_) [12]. Due to the anoxic conditions required and availability of suitable electron donors, denitrification is not limited in the sediments of shallow water ecosystems [13]. Although anammox has been reported to account for up to 67% of N_2_ production in marine shelf sediments [14, 15] and 0%– 13% in limnic systems [16, 17, 18], the importance of this process in shallow freshwater systems is not well defined [17]. Both processes depend on the supply of NO_3_^-^ or NO_2_^-^ produced in the overlying oxic sediment layer or in adjacent watersheds. Nitrate reduction to N_2_ also depends on direct competition with NO_3_^-^ reduction to NH_4_^+^ by another anaerobic process, dissimilatory NO_3_^-^ reduction to NH_4_^+^ [19, 20], in addition to competition with NO_3_^-^ assimilation by algae, specifically benthic microalgae. Thus, the extent to which surplus inorganic N in sediments is removed or recycled in the sediment or is released into the water column as NH_4_^+^ or NO_3_^-^ depends on the magnitude and coupling of nitrification, denitrification, anammox, and dissimilatory nitrate reduction to ammonium (DNRA), as well as the NO_3_^-^ assimilation driven by microorganisms.

In NO_3_^-^ limited environments, DNRA is expected to be favored in competition with denitrifiers [21, 22, 23]. However, denitrification would be expected to be more prevalent if the electron donor, such as organic carbon (C), is limited because this process releases more energy per mole of oxidized carbon [24, 25]. Another important parameter determining which process is favored may include the metabolic versatility of the bacteria involved. Denitrifiers are facultative anaerobic bacteria, whereas the metabolic alternatives for most DNRA bacteria are fermentation or DNRA coupled to chemolithoautotrophic sulfur oxidation [26, 27]. Therefore, the efficiency and dominance of each N process also depends on the environmental conditions, which in turn influence the metabolic processes of the microorganisms present [22, 25]. A comparison across a wide range of habitats confirms that the denitrification/DNRA ratio correlates positively with a high availability of NO_3_^-^ and/or O_2_ relative to carbon [28, 29]. Recently, Kraft et al. (2014) [23] showed that under high NO_3_^-^ conditions, the microbial generation time, supply of nitrite (NO_2_^-^) relative to NO_3_^-^, and C/N ratio are the key environmental factors that control the fate of NO_2_^-^ to denitrification or DNRA. Therefore, competition among these processes can vary with environmental characteristics and can control the availability and amount of reactive N.

Evaluating N transformations in tropical aquatic systems with wide variability can provide important information on the regulatory mechanisms and dynamics of the sediment and water N cycle, including the extent of N limitation, risks of N excess and environmental controls, knowledge that is important because the N cycle in tropical ecosystems remains unclear. In our study, we measured certain N transformations as actual sediment denitrification rates and the relationship among potential processes such as O_2_ consumption, nitrification, denitrification, anammox and estimated DNRA. We also assessed the relative importance of these processes in shallow water environments characterized by low water NO_3_^-^ concentrations and diverse water chemistry processes, a complex sediment biological structure, and varying degrees of organic matter decomposition, as well as the presence or absence of sediment algal and microalgal mats. These analyses included different types of tropical shallow water ecosystems in the coastal zone.

## Materials and Methods

### Locations and sampling

No specific permission was required to collect water, sediment and limnological parameters data in Restinga de Jurubatiba National Park (22°–22° 30’ S and 41° 15’–42° W) from 2000 and 2001. In addition, the field and laboratory studies did not involve any biological species.

In September 2000, sediment samples were collected for determining actual denitrification rates in intact sediment cores using the isotope pairing technique [30] described by Enrich-Prast et al. (2015) [4]. In September 2001, sediment was collected to measure the potential activity of denitrification, nitrification, anammox, and O_2_ consumption in homogenized slurries. Potential DNRA activity was not measured, but was estimated. In both years, sediment samples were collected from 12 lagoons, both salt and hypersaline, inside or near the Restinga de Jurubatiba National Park (northern Rio de Janeiro/Brazil), a conservation area of 14,838 ha on the Atlantic coastal plain and encompassing sandplains, coastal lagoons, and shrub vegetation. More information about the 12 lagoons is available in Caliman et al. 2010 [31]. The area of northern Rio de Janeiro is characterized by a tropical sub-humid/humid climate with an annual precipitation of 1,165 mm, a mean summer temperature of 25°C and a mean winter temperature of 19°C [32]. During sampling, the temperatures *in situ* were 26°C– 29°C, and the water used during experiments was always well aerated (50%– 150% of air saturation), with no significant stratification or tidal effects. The study locations were selected to coverthe maximal variability in terms of nutrient level, salinity, pH, algal and microalgal colonization, and the dominant types of primary producers. (Table 1).

**Table 1:** Physico-chemical characteristics of the water column and sediment from the lagoon collection sites from September 2001.

Location	Salinity	Depth	chl-*a*	NO_3_^-^	NH_4_^+^	TN	TP	pH	Sediment
	(us)	(m)	(μg L^-1^)	(μM)	(μM)	(μM)	(μM)		
**Pires**	36	1	50.0	2.0	3.8	30.5	6.5	8.43	Algal mat
**Imboassica**	30	1	122.5	0.6	3.9	61.6	2.7	8.03	Mud with detritus
**Encantada**	40*	0.8	4.5	0.3	2.6	121.0	1.8	4.0	Microbial mat
**Piri-piri**	60*	0.2	2.5	1.7	0.0	53.0	1.7	7.96	Microbial mat
**Preta**	40*	2	11.2	2.5	11.7	43.1	1.3	8.39	Microbial mat
**Reservoir**	0	1.5	3.0	2.9	3.5	54.8	3.6	7.86	Mud with detritus
**Carapebus**	3	2.5	5.6	0.9	8.6	67.0	0.6	7.70	Silt with macrofauna
**Cabiúnas**	8	2.5	11.5	0.6	1.6	55.6	0.4	7.30	Sand with macrophytes
**Menina**	50*	0.5	7.6	1.6	2.7	60.9	4.9	8.10	Microbial mat
**Paulista**	35	3	4.8	1.1	2.4	48.5	0.5	6.18	Sand with macrophytes
**Comprida**	0.1	1.5	8.6	1.9	2.8	64.5	0.5	5.54	Peat and sand
**Iodada**	1	0.5	9.7	2.1	3.4	73.6	6.0	6.02	Sand and detritus

*Hypersaline lagoons

Most of the sites were lagoons separated from the sea by sand barriers, with a salinity ranging from 0 to 60 depending on seawater intrusion, freshwater input, and evaporation. More detailed information can be found in Suhett et al. (2007) [33]. The sediment C content (mg C g^-1^ dry weight^-1^) was obtained for only two lagoons (Imboassica and Cabiúnas, at 7.9 and 5.8, respectively) [4]. The typical vegetation found in the restinga area produces freshwater lagoons with poor nutrient drainage and acidic, humic water; in contrast, the lagoons receiving water from local inland settlements are nutrient rich with an abundance of phytoplankton [34]. The hypersaline lagoons are dominated by benthic microalgal mats composed of microphytes and sulfuric bacteria (Table 1).

For sampling at each location, we selected the deepest area, where net organic matter mineralization was expected to be relatively great due to a higher sedimentation rate for that given ecosystem. Seven plexiglas cores (15.8 × 3.6 cm) with intact sediment were collected manually. An improvised long stick (3.0 m) was used for water depths <1.0 m, whereas a vent stopper was used for water depths >1.0 m. Special care was taken to minimize sediment disturbance during manual or stick sampling. After sampling, O_2_ was measured at the top of the sediment using an O_2_ microsensor (Unisense, Denmark). Water was collected near the sediment surface using a Van D’orn bottle; the water samples were kept aerated and at the in situ temperature. The samples were then transferred to 1 L bottles and transported within three hours at low temperature to the laboratory at the NUPEM field station. Visual observations of the water, sediment structure, fauna, and vegetation were performed in the field laboratory.

Using magnifying glasses, we determined the absence or presence of algae on the top of the sampled sediment. The presence of an algal or microalgal mat on the sediment was noted for Pires, Encantada, Piri-Piri, Preta, and Menina lagoons (Table 1).

### Potential oxygen consumption

At each site, subsamples of 0.5 cm^3^ homogenized surface sediment were placed in five 6.7 mL gas-tight vials (exetainers, LabCo). To continuously mix the sediment with air and minimize the effect of chemical O_2_ consumption on O_2_ measurements, the vials were constantly rolled with adapted rolling mill equipment for a minimum of 2 h. Thereafter, the vials were filled completely with fully aerated water from each sampling site, closed, maintained in the dark at 28°C, and manually mixed for 1 minute every 10 minutes. The oxygen concentration in the slurries was repeatedly measured with a fast response needle minisensor (<15 s) after 0 min, 15 min, 30 min, 1 h, and 2 h until a significant linear decrease was observed (>10% depletion). During the short time-interval measurements, we placed an exetainer lid on the tip of the needle so that the slurry vial remained closed (no contact with air); as lid replacement by the needle with another lid took less than 2 s, contact between the slurry and air was limited. To evaluate air contamination, we performed several measurements with slurries at different O_2_ concentrations; we observed no significant O_2_ contamination during this procedure, indicating that the procedure did not affect the O_2_ concentrations in the slurries, as O_2_ exchange between the slurry and atmosphere was negligible during lid replacement. The detection limit of the O_2_ sensor was <0.1% saturation. Oxygen consumption per cm^3^ of wet sediment was estimated based on changes in O_2_ over time at a constant salinity and temperature.

### Relative potential nitrification

To measure potential nitrification as a fraction of total biological O_2_ consumption, the method described by Ottosen et al. (1999) [35] was used, with some modifications. Essentially, the sediment was incubated with surplus ^15^NH_4_^+^ at a defined initial O_2_ concentration; then, the amount of ^15^NO_3_^-^ produced and later denitrified to N_2_ was analyzed. To measure O_2_ consumption, the first five vials were prepared as described above, and aeration was maintained until incubation. Then, 2 mL of aerated water from the site was added, followed by 0.1 mL of 33.5 mM ^15^NH_4_^+^ to yield a final concentration of approximately 500 μM; a final concentration of 50 μM was reached after adding water plus 0.1 mL unlabeled 3.35 mM NO_3_^-^. Immediately after this step, the vials were filled to maximum capacity with aerated site water and sealed. One vial without any additions was used as a background reference. The relative potential nitrification was calculated from the amount of ^15^N-N_2_ produced, the initial O_2_ concentration, and the denitrification fraction of total NO_3_^-^ reduction as obtained from the denitrification-DNRA assay. The calculation was based on complete oxidation to NO_3_^-^; however, because it is possible that some NH_4_^+^ was oxidized to NO_2_^-^ before all of the O_2_ was depleted. O_2_ consumption due to nitrification in the vials may be overestimated by up to 33% [35].

### Actual denitrification

After sampling and transport to the field laboratory, undisturbed sediment cores from each lagoon were independently arranged in small vessels covered with lagoon water and aerated at the *in situ* temperature in the dark for 2 h for stabilization. ^15^NO_3_^-^ was then added in a similar procedure as described by Enrich-Prast et al. (2015) [4]. Denitrification rates were obtained using the isotopic pairing technique proposed by Nielsen (1992) [30] after addition of 1 mL of ^15^NO_3_^-^ (5 mM) to each sediment core; according to Dalsgaard et al. (2000) [36], this would yield a concentration of approximately 100 μM in the water column. Denitrification rates were obtained by assuming a random mixture of added ^15^NO_3_^-^ and ^14^NO_3_^-^ in the water and that were produced in the sediment by nitrification. The formation of ^29^N_2_ (^14^N^15^N) and ^30^N_2_ (^15^N^15^N) by the end of the incubation period was used to calculate the actual denitrification rates, coupled nitrification–denitrification (Dn) and denitrification from the NO_3_^-^ present in water column (Dw) fractions [30].

### Potential denitrification and estimated DNRA

The vials used for potential O_2_ consumption measurements were again used to determine NO_3_^-^ reduction after the addition of ^15^N-NO_3_^-^. To ensure that O_2_ was completely consumed, prior to incubation, the vials were closed for 24 h after the time that all O_2_ should theoretically have been consumed. This procedure was previously verified in parallel incubations. Four of the vials were supplied with 0.1 mL of a de-aerated solution of 3.35 mM ^15^NO_3_^-^ to yield a final concentration of 50 μM. The vials were also supplied with 0.1 mL of unlabeled NH_4_^+^ to a final concentration of 500 μM to ensure that only NH_4_^+^, and not NO_3_^-^, would be assimilated. The last vial without any additions was used as a reference. The vials were incubated for additional time (this period exceeded 48 h for slurries with low potential O_2_ consumption) to ensure that all NO_3_^-^ was reduced and preserved by the addition of 0.1 mL of saturated ZnCl_2_ solution. Potential denitrification as a fraction of total NO_3_^-^ reduction was calculated from the recovery of added ^15^NO_3_^-^ as ^15^N_2_. DNRA was roughly estimated by assuming that the ^15^NO_3_^-^ not recovered as ^15^N_2_ would be allocated to DNRA. Because ^15^NO_3_^-^ transformation to ^15^NH_4_^+^ was not directly measured, this metodology enabled only approximated estimates of DNRA rates; as such, these DNRA estimates should be evaluated cautiously. In this study, we assumed that all ^15^NO_3_^-^ was transformed into ^30^N_2_ (measured) and ^15^NH_4_^+^ (estimated). Although some of the ^15^NO_3_^-^ may have been transformed into other byproducts, such as NO_2_^-^, nitric oxide (NO), and nitrous oxide (N_2_O), during the incubation, it is unlikely that N byproducts would accumulate in an active anoxic slurry; therefore, we assumed that all molecules were consumed before the addition of ZnCl_2_.

### Potential anammox

Vials for potential anammox incubations were prepared in the same manner as described above for potential O_2_ consumption and left closed until all the O_2_ and NO_3_^-^ were completely depleted (this period exceeded 48 h for some slurries). Incubation was initiated by injecting 0.1 mL of a de-aerated 33.5 mM ^15^NH_4_^+^ stock and 0.1 mL of a de-aerated 6.7 mM ^14^NO_2_^-^ stock. The resulting ^15^NH_4_^+^ concentration of 500 mM was designed to be multiple orders of magnitude higher than the background concentration of ^14^NH_4_^+^, which could then be ignored in the calculations. The initial concentration of ^14^NO_2_^-^ was 100 μM. The vials were incubated for a minimum of one day to ensure that all the NO_2_^-^ was reduced, and the vials were analyzed for ^15^N-N_2_ as described for the potential denitrification assay. Potential anammox as a fraction of total NO_2_^-^ reduction was calculated from the recovery of ^14^NO_2_^-^ as ^15^N^14^N-N_2_. The vials were incubated for additional time (this period exceeded 48 h for some slurries) to ensure that all the NO_3_^-^ was reduced and then preserved with the addition of 0.1 mL of saturated ZnCl_2_ solution.

### Analytical and statistical methods

Water temperature and salinity were measured *in situ* and in the field laboratory using an oxymeter (YSI-55). pH was measured using a pH meter (Analion, BR). Water samples were transported at 4°C to the field laboratory within 3 h, filtered through 47-mm GF/F filters, and immediately frozen. Samples of the unfiltered water, filtered water, and filters were frozen separately for later analysis of total and dissolved N and P as well as chlorophyll-*a* [37]. In all exetainers used for potential nitrification, denitrification, and anammox incubations, 1 mL of slurry was replaced with 1 mL of He for extraction of N_2_. A 250 μL subsample of gas was injected into a GC-MS system (Isomass Co.) at the University of Aarhus for analysis of excess ^15^N-N_2_, as described by Dalsgaard et al. (2000) [36]. Denitrification rates, potential O_2_ consumption, and potential and estimated N processes did not demonstrate a Gaussian distribution (Shapiro-Wilk normality test) and the Kruskal-Wallis test followed by Dunn’s post test, which were both evaluated at 5% level, were used to compare each N process rate at all sites. We performed Spearman’s correlation between each N process rate and the potential O_2_ consumption and the limnological parameters (water temperature, depth, salinity, pH, chlorophyll-*a*, NO_3_^-^, NH_4_^+^, TN and TP) for each site. The Mann-Whitney U test (p < 0.05) was used to compare actual denitrification with the Dn and Dw fractions. All analyses were conducted using GraphPad Prism (Version 4, for Windows, GraphPad Software, San Diego California USA).

## Results

**Fig 1:**
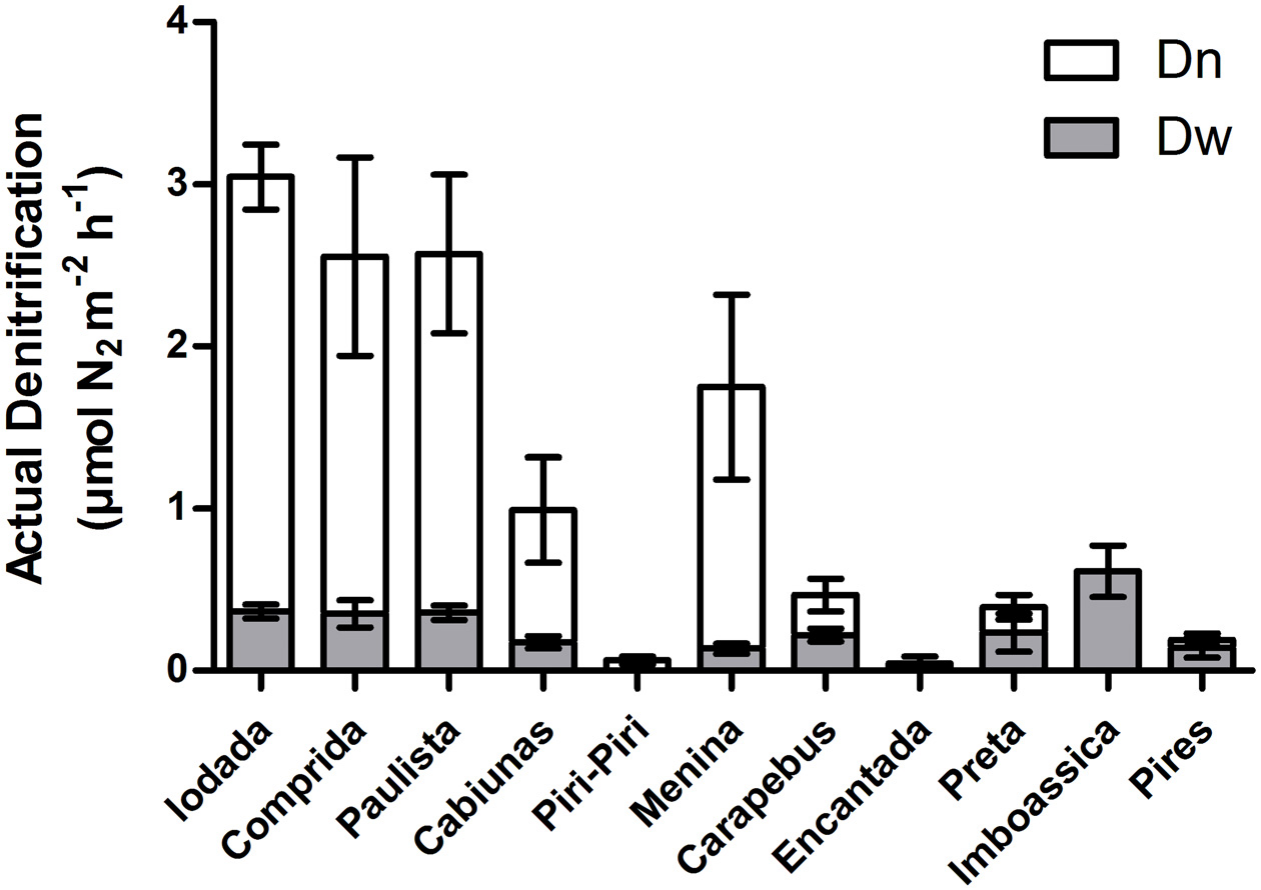
Actual denitrification and Dn (white bars) and Dw (shaded bars) rates (μmol N_2_ m^-2^ h^-1^, n = 5, average ± standard error) measured in intact sediment cores of study site lagoons.

The actual denitrification rates were low in all the studied environments, never reaching values higher than 3.1 μmol N_2_ m^-2^ h^-1^ (Fig 1). The highest rates were found in Iodada, Comprida and Paulista (3.04 ± 0.24, 2.5 ± 0.7 and 2.6 ± 0.5 μmol N_2_ m^-2^ h^-1^, respectively) lagoons. Additionally, actual denitrification was insignificant at some lagoons, such as Piri-Piri and Encantada (0.063 ± 0.025 and 0.046 ± 0.05 μmol N_2_ m^-2^ h^-1^, respectively).

**Fig 2:**
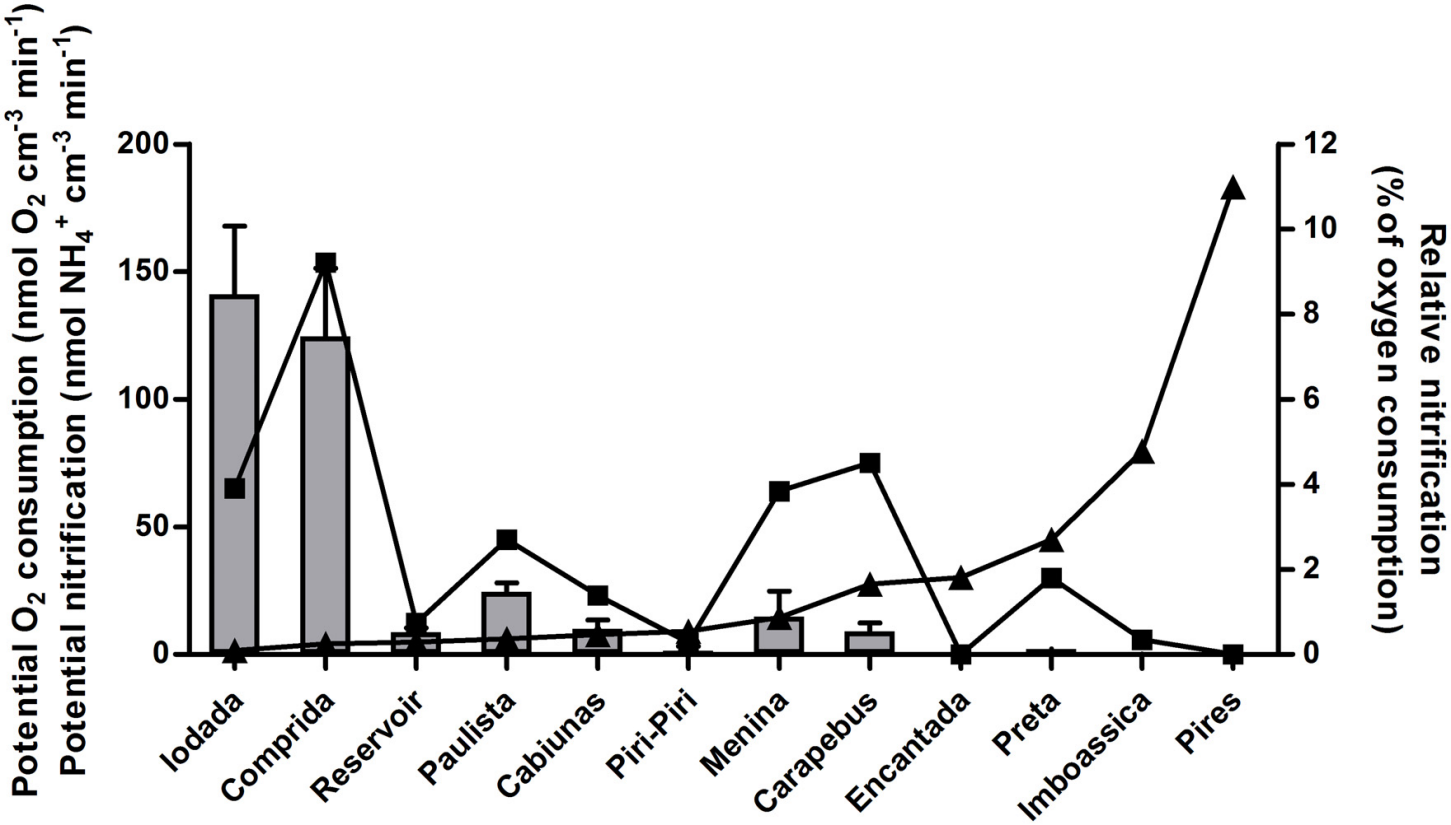
Evaluation of NO3- production and organic matter mineralization at each site sampled according to the following: rates of potential O_2_ consumption (line with an open circle, nmol O_2_ cm^-3^ min^-1^), potential nitrification (line with a black square, nmol NH4+ cm^-3^ min^-1^) and relative nitrification (gray bars, % of O_2_ consumption; average ± standard error).

Coupled nitrification–denitrification (Dn) prevailed over the denitrification of NO_3_^-^ present in the water column (Dw) at Iodada, Comprida, Paulista, Cabiúnas and Menina, demonstrating that the NO_3_^-^ produced by nitrification is an important source in these lagoons compared with the NO_3_^-^ from external sources. The potential O_2_ consumption rates at these locations were lower than at other lagoons, with a maximum value of 14.4 ± 1.3 μmol O_2_ m^-2^ h^-1^ (average ± standard error; Fig 2), which maintains available O_2_ consumption by nitrification.

Overall, Dw was very low and did not exceed 0.61 μmol N_2_ m^-2^ h^-1^. However, the NO_3_^-^ in water was the main source for denitrification in certain lagoons (Preta, Imboassica and Pires), with a Dn value lower than that of Dw. These findings may be associated with the high potential O_2_ consumption in those sediments. The high value at Pires [the highest value of 183.3 ± 9.3 μmol O_2_ m^-2^ h^-1^ (average ± standard error; Fig 2)] would inhibit nitrification activity.

Potential O_2_ consumption rates were significantly negatively correlated with potential denitrification, potential nitrification and potential anammox rates; significantly positively correlated with estimated DNRA, whereas no correlation with actual denitrification was observed. None of the potential or estimated processes showed a correlation with the measured limnological parameters.

Potential O_2_ consumption varied greatly among the sites (Fig 2). To complete any rapid chemical O_2_ consumption driven by the oxidation of free ferrous iron or sulfide, the samples were pre-aerated prior to measurements. Thus, the measured potential O_2_ consumption rates should be predominantly based on the biological oxidation of organic matter, though some oxidation of particulate iron-sulfur compounds may have occurred. The potential O_2_ consumption rates varied by more than two orders of magnitude from 1.5 to 180 nmol cm^-3^ min^-1^ (Fig 2). The highest rates were found in the microalgal mat habitats (Pires, Preta, and Encantada) and the eutrophic estuary that formed the Imboassica lagoon, whereas the lowest activity was recorded in the two humic freshwater lagoons.

The relative potential nitrification rate varied from 0.01% to 9% of the potential O_2_ consumption rate (Fig 2) and was significantly higher (Mann-Whitney U, p<0.05) in environments with low potential O_2_ consumption rates, exihibiting values between 0 and 1 nmol O_2_ cm^-3^ min^-1^ [38].

**Fig 3:**
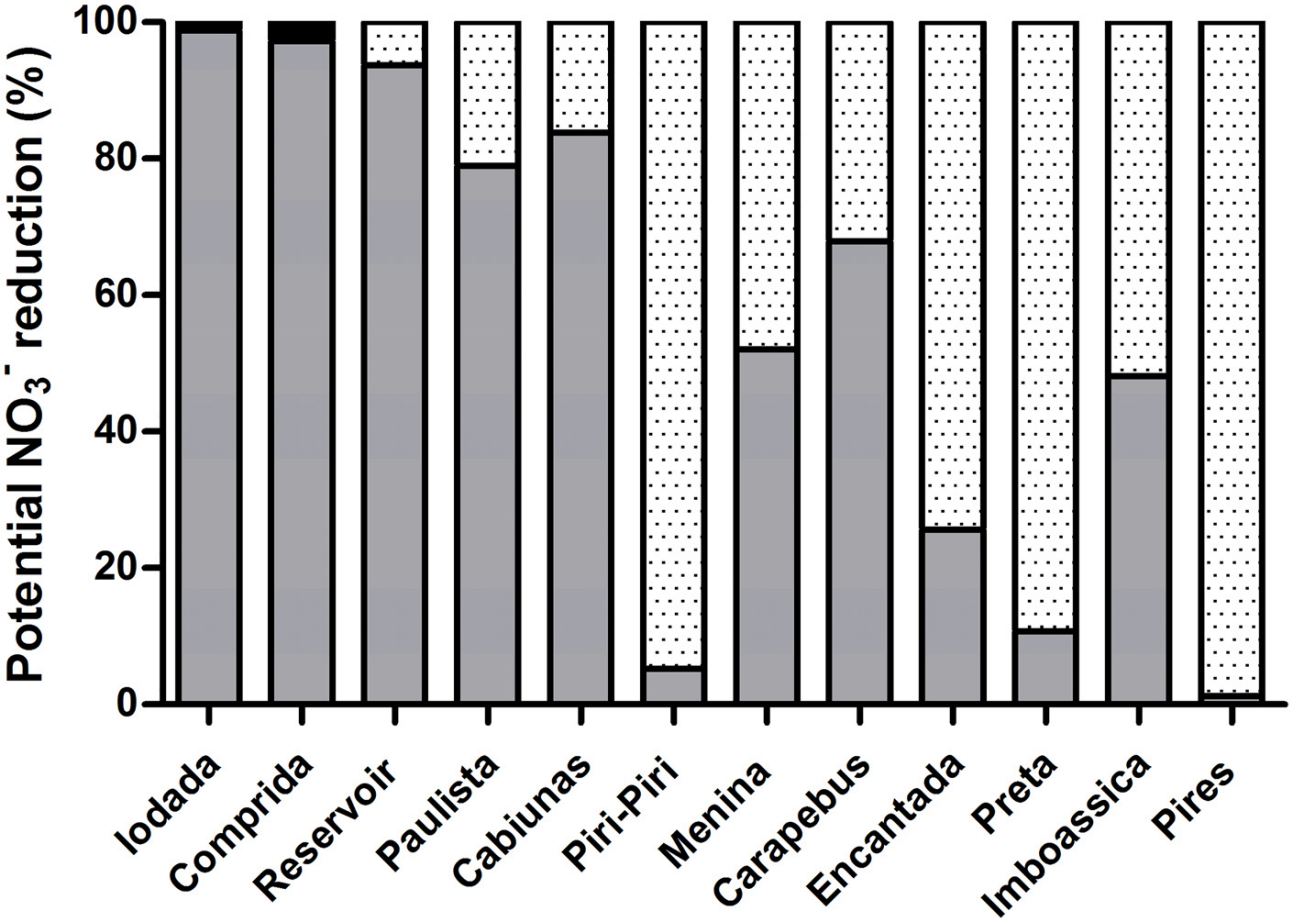
N cycle anaerobic processes in sediment slurries based on NO3- reduction. Left axis represents the percentage of N reduction in each process: potential anammox (black bars), potential denitrification (dark gray bars) and estimated DNRA (light gray bars).

The reduction of NO_3_^-^ clearly shifted from almost 90%–95% of potential denitrification at sites with low potential O_2_ consumption rates (Iodada, Comprida, Reservoir, Paulista, Cabiúnas, Piri-Piri and Menina) to values between 75% and 98% of estimated DNRA (Fig 3) at sites with higher potential O_2_ consumption rates: Carapebus, Encantada, Preta, Imboassica and Pires (Fig 2).

Potential anammox was detectable only at sites with low potential O_2_ consumption rates (between 0 and 1 nmol O_2_ cm^-3^ min^-1^). Although production of ^30^N_2_ may have occurred via a combination of DNRA and anammox, leading to an overestimation of the potential denitrification rates (Fig 3), there were only two environments where anammox accounted for more than 1% of NO_3_^-^ reduction (Iodada at 3.4% and Comprida at 1.1%). In all other environments, potential anammox was responsible for <0.1% NO_3_^-^/ NO_2_^-^ reduction (Fig 3). These data suggest that overestimation may account for a maximum of 3% and 1% of the expected potential denitrification attributed to the estimated DNRA at the Iodada and Comprida lagoons, respectively.

**Fig 4:**
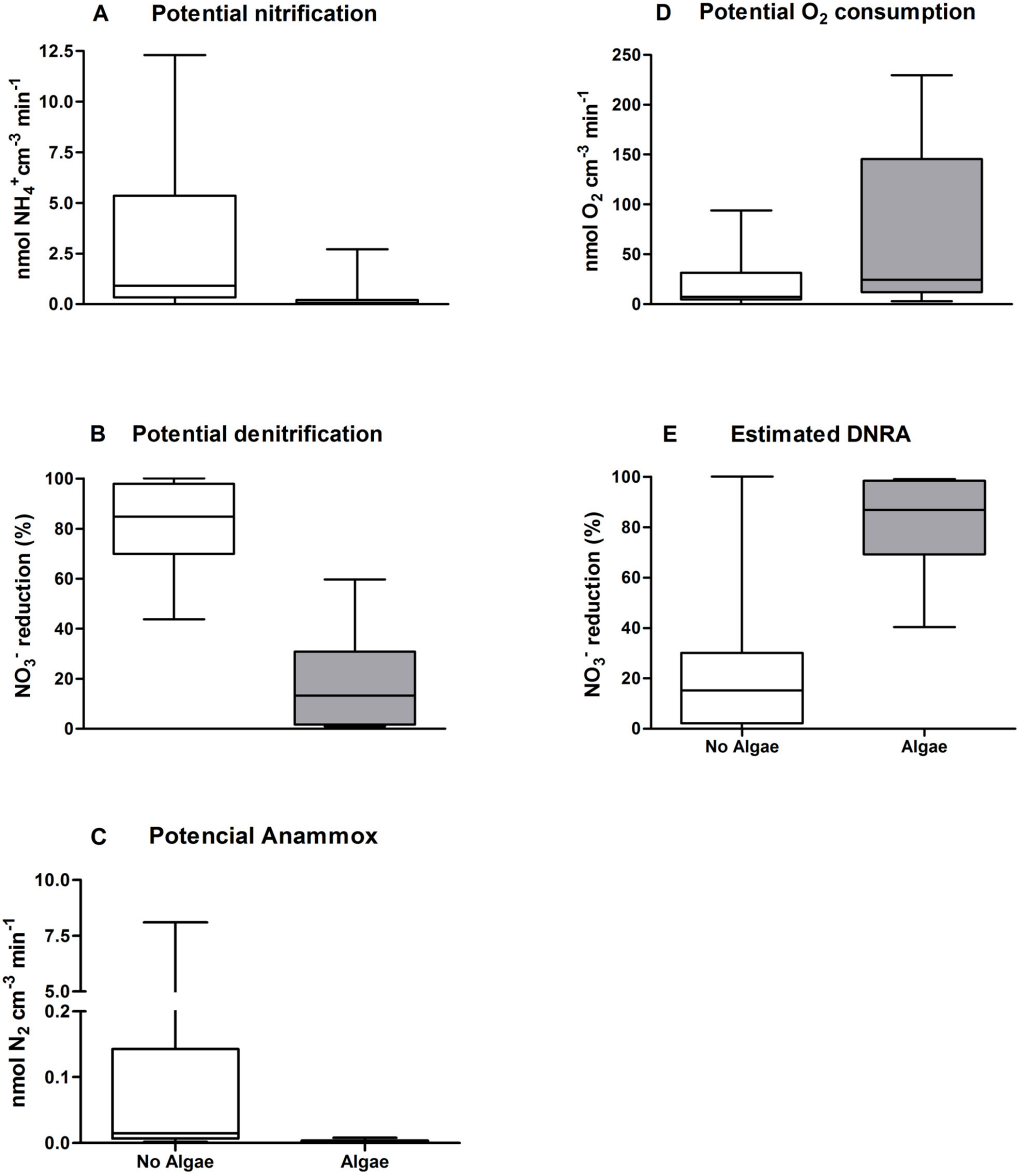
Influence of algal and microalgal mat on the following processes. A) Potential nitrification, B) Actual denitrification, C) Potential anammox, D) Potential O_2_ consumption, E) Estimated dissimilatory nitrate reduction to ammonium (DNRA). Box and whiskers, min and max. The presence of algal and microalgal mat had a highly significant influence over all studied processes (n = 6; p<0.001, Mann- Whitney U), except for potential O_2_ consumption (D), where the difference was significant (n = 6; p<0.05, Mann- Whitney U).

Those sediments colonized by algal and microalgal mats showed significantly lower (Mann-Whitney U, p<0.001) potential nitrification (Fig 4A), potential denitrification (Fig 4B), and potential anammox (Fig 4C) rates as well as significantly higher potential O_2_ consumption (Fig 4D) and estimated DNRA (Fig 4E; Mann-Whitney U, p<0.05) rates. Potential nitrification and potential anammox were almost absent in sediments colonized by algal and microalgal mats.

## Discussion

### Potential aerobic processes

*In situ* O_2_ consumption is limited by diffusion or advection of O_2_ from the water column. Additionally, extension of the oxic zone seems to decrease concomitantly with a rise in aerobic microbial intensity, and the role of anaerobic degradation increases relative to aerobic mineralization [11]. As O_2_ concentrations in the water column close to the sediment were near air saturation levels in all studied ecosystems and O_2_ was consistently available near the sediment surface at all sampling locations (data not shown), potential O_2_ consumption rates were used as a proxy for the degree of organic matter turnover, as also used in previous studies [39].

The release of organic matter as a result of photosynthetic activity is another explanation for the high sediment potential O_2_ consumption rates observed in some studied lagoons. Organic matter exudation and the availability of high quality organic matter due to decaying benthic algal and microalgal mats would dependente positively on N and phosphorus scarcity [40], as has been shown for sediments colonized by these organisms [41]. Indeed, thee organic matter produced can stimulate heterotrophic activity, which is reflected in higher potential O_2_ consumption rates (Fig 4D). The benthic algal and microbial mats present in some of the studied sediments may explain the different processes observed among the studied lagoons (Fig 4). Microalgae compete with microorganisms for inorganic N, mainly NH_4_^+^, and may inhibit the development and growth of nitrifying bacteria, thereby regulating denitrification due to low NO_3_^-^ availability [42, 43]. Such inhibition in the presence of algae or microbial mats can be associated with low potential nitrification and potential denitrification rates in sediments (Fig 4A and 4B).

Risgaard-Petersen (2003) [42] observed a similar result for nitrification, oxygen consumption, and denitrification in a range of temperate sediments colonized by microalgal mats. The author concluded that photosynthetic microorganisms compete for inorganic N, inhibiting the development and growth of nitrifying bacteria and thereby regulating denitrification, a result further confirmed by Nizzoli et al. (2014) [44].

Sediment nitrification is commonly limited by the availability of NH_4_^+^ and O_2_ or by the abundance of nitrifying bacteria [44], which may indicate that NH_4_^+^ is limiting in sediments harboring algal and microbial mats. However, O_2_ availability may also become a relevant factor because of high aerobic potential O_2_ consumption rates during photosynthetic activity. *In situ* nitrification activity is also regulated by the depth of O_2_ penetration and density of bacterial populations. Furthermore, the population density is reflected by the specific potential nitrification rate, which can be derived by multiplying the measured specific O_2_ consumption rate by the relative contribution of nitrification [35]. Surprisingly, this rate does not correlate with the overall activity, which may indicate that some undisclosed density dependent mechanisms, such as predation, can control the population density of nitrifying bacteria in the studied environments [45].

The potential nitrification rates found in this research were very low, approximately one order of magnitude lower than previously reported [35, 46–47]. This difference could be attibuted to the methodology employed because sediment potential nitrification was measured at a small scale and did not evaluate the potential NO_2_^-^ that may be produced as a final product instead of NO_3_^-^. Based on the low potential rates obtained, we roughly estimated the extent to which the nitrification capacity could possibly match the *in situ* production of NH_4_^+^ due to mineralization. If we assume that the C/N ratio of net mineralization in the sediment varies from 8 to 30 depending on the source of organic matter and that the oxidation of one C requires one oxygen, whereas one NH_4_^+^ requires two oxygen molecules, it can be estimated that potential nitrification must represent a minimum of 6%– 25% of oxygen consumption if all net NH_4_^+^ production is oxidized [48]. Only those environments with lower potential O_2_ consumption rates, Iodada and Comprida, matched these criteria, with a relative nitrification rate of 9% (Fig 2). In the other ecosystems, it is expected that most of the net mineralized N will not be oxidized but rather will leave the sediment as NH_4_^+^. This surplus of NH_4_^+^ is even more pronounced when anaerobic NH_4_^+^ production by mineralization and DNRA is taken into account.

### Sediment actual denitrification

The low sediment actual denitrification rates in all the studied environments were up to two orders of magnitude lower than the average estimated for coastal lagoons [1, 39, 49], confirming that the denitrification rates in tropical environments are relatively low in relation to temperate environments. Moreover, the environmental conditions were markedly different among the lagoons, yet the actual denitrification rates were low at all sites. The main explanation for these low actual denitrification rates may be the low NO_3_^-^ concentrations in the water column. A recent study also found low denitrification rates at two of the studied environments (Cabiúnas and Imboassica) [4] and these low rates were attributed to the low NO_3_^-^ concentration in the water column. However, despite the low rates, a difference in actual denitrification between sites with low and high potential O_2_ consumption was found (Figs 1 and 2) because O_2_ availability regulates denitrification activity; in addition, the predominance of denitrification based on nitrate from coupled nitrification-denitrification (Dn) positively correlated with potential O_2_ consumption. The highest actual denitrification rates were observed in sediments with low potential O_2_ consumption where Dn was dominant because O_2_ was available for nitrification and supplied NO_3_^-^ for denitrification. Denitrification based on nitrate from the water column (Dw) was similar and low in all of the studied lagoons, representing the only denitrification activity in those lagoons with high potential O_2_ consumption.

### Potential anaerobic processes

The sediment organic matter functions as a substrate that provides food for fauna, aiding in its stability, and storage for carbon and nutrients [50]. The mineralization of sediment organic matter (i.e., O_2_ consumption) in shallow ecosystems is often attributed to the release of labile forms of C and N by microbial biomass [51].

In general, DNRA prevails in sediments with high C/N ratios [21, 23], representing a large amount of organic matter and NO_3_^-^ limitation [20], which occurs during electron transfer via DNRA process. DNRA microorganisms transfer eight electrons per mole of NO_3_^-^ reduced, whereas only five electrons are transferred during denitrification [21]; therefore, more organic matter is necessary to supply a higher quantity of electrons for DNRA. Those sites with high organic matter mineralization represented here as high potential O_2_ consumption were the same lagoons with estimated DNRA (Figs 2 and 3). This finding explains the dominance of denitrification at these sites because denitrification is favored in anoxic microzones [43, 52], as demonstrated at the Cabiúnas site where Enrich-Prast et al. (2015) [4] observed relatively higher denitrification rates in comparison with the Imboassica lagoon. This finding is likely associated with coupled nitrification-denitrification, which requires adjacent oxic and anoxic zones. As discussed above, this coupling provides more NO_3_^-^ than other sources to the same lagoons where potential O_2_ consumption is low, and potential denitrification is high. In contrast, the opposite situation of low potential nitrification was observed in sediments with high estimated DNRA activity.

pH can also regulate competition between denitrification and DNRA, as pH values above 6.5 favor DNRA bacterial reduction of NO_3_^-^ in sediments [53]. Concomitant with other environmental factors, pH seems to control the estimated DNRA at Iodada, Comprida and Paulista sites, where the pH values were lower than 6.5 (Table 1). Indeed, as estimated DNRA was absent, potential denitrification was the main process in these locations (Fig 3).

Three outliers that did not fit the denitrification/DNRA pattern were observed (Fig 3). The high O_2_ consumption values at Piri-Piri suggest the occurrence of a higher denitrification/DNRA ratio than that observed. The sediment from Piri-Piri was classified as a highly active microbial mat, and it likely that the potential O_2_ consumption rate was underestimated for this location. This may have occurred because O_2_ consumption was measured without light and in the field, and O_2_ production during photosynthesis may promote an increase in respiration rates [42]. The other outliers include the two largest lagoons, Carapebus and Imboassica, which yielded higher denitrification/DNRA ratios than predicted. Because these lagoons are large and shallow, strong winds resuspend the sediment more frequently, thereby stimulating aerobic mineralization [54] at the expense of anaerobic mineralization. This phenomenon can promotes na increase in denitrification compared with sediments that may present limited O_2_ diffusion.

In general, potential anammox was observed in zones where NH_4_^+^ and NO_3_^-^/NO_2_^-^ are available [22], such as the water-sediment interface, which is primarily regulated by NO_3_^-^/NO_2_^-^ [55]. If the presence of anoxic zones that also harbor NH_4_^+^ and NO_3_^-^/NO_2_^-^ were the only requirement for the establishment of these bacteria, anammox would be expected to be occuring in virtual sediments. However, because the process is relatively less importante in high-organic carbon sediment, anammox can be indirectly regulated by organic matter [22], as we observed in the studied lagoons. Low or absent potential anammox rates may also be attributed to a higher generation time among the microorganisms responsible [56–58], which makes the sustainability of anammox bacterial populations dependent on a long-term stable micro-environment. Those conditions are, to some extent, fulfilled in environments with lower potential O_2_ consumption rates, where the potential anammox zone would be wider and deeper in the sediment. This may explain why potential anammox was detected at Carapebus (0.013 ± 0.01%; average ± standard error). Compared with our potential anammox results, Crowe et al. (2012) [59] found a very high contribution of anammox in intact core sediment and slurry (32.9% and 67%, respectively). The high contribution observed by these authors was linked to NO_3_^-^ in the overlying water and sediment organic matter, which explains the very low anammox contribution because the NO_3_^-^ concentration in the studied lagoons was low.

Salinity can be highlighted as another limnological parameter limiting anammox, as shown by Trimmer et al. (2003) [60] in the Thames river estuary (UK). These authors found a gradient of anammox that increased together with salinity across the river, though our data did not show any influence os salinity because the highest contribution of potential anammox was observed only in two lagoons with very low salinity. More studies are needed before broad generalizations concerning the distribution and importance of anammox in coastal environments can be made. However, our results do suggest that sediment O_2_ consumption may have a prevailing role over other environmental factors, specifically salinity.

### Proposed model for denitrification and DNRA

**Fig 5:**
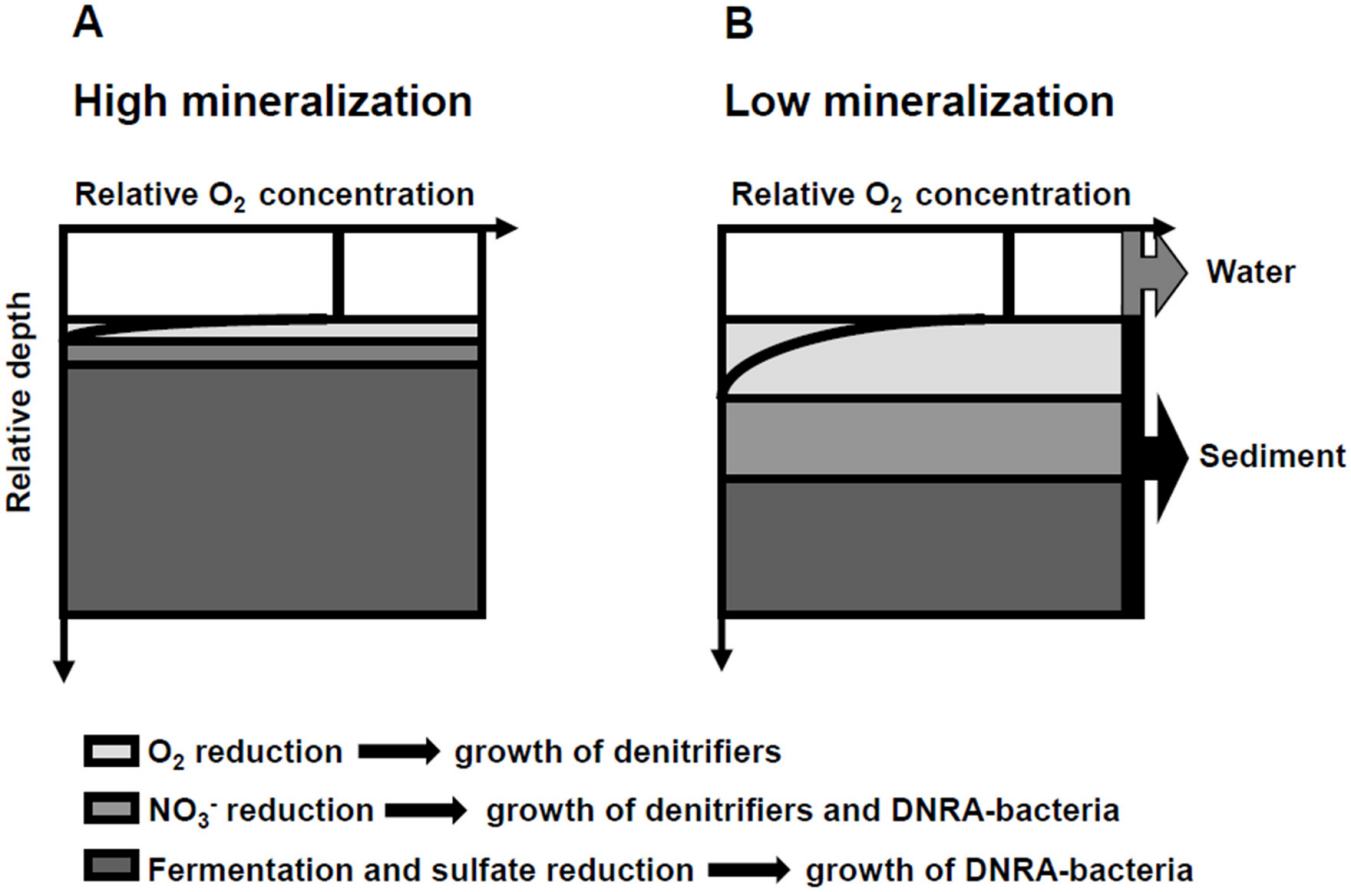
Suggested model for the growth and development of denitrification and dissimilatory nitrate reduction to ammonium (DNRA) bacteria in sediments with high and low mineralization rates. The sediment is divided into oxic (light gray), oxic/anoxic (middle gray) and anoxic (dark gray) sediment zones.

Considering that O_2_ penetration into the sediment is usually regulated by the mineralization rate [48], we propose a theoretical model based on sediment O_2_ penetration to explain the growth, activity, and distribution of microorganisms responsible for denitrification and DNRA in sediments with low NO_3_^-^ and NO_2_^-^ availability (Fig 5).

Environments with high mineralization rates have lower O_2_ penetration into the sediment than environments with low mineralization rates and vice-versa [48]. Therefore, this model presents two scenarios that occurs in sediments with high mineralization rates (Fig 5A) and in those with low mineralization rates (Fig 5B), excluding sediment resuspension and bioturbation.

In general, a thin oxic layer is expected to exist, immediately below the bhentic boundary layer, in sediments with high mineralization rates (Fig 5A), where O_2_ is consumed during the mineralization of aerobic organic matter. When O_2_ is present, denitrifiers in this thin layer can survive by performing aerobic heterotrophy using O_2_ as an electron acceptor as these organisms are obligatory anaerobes, DNRA activity would be absent. Oxic and anoxic conditions may alternate in a second, thin intermediate zone, and the growth and activity of both denitrifiers and DNRA bacteria can coexist.

A different scenario is observed in sediments with low organic matter mineralization rates (Fig 5B). Lower O_2_ consumption would usually lead to higher sediment O_2_ penetration, creating a larger oxic layer and leading to the dominance of denitrifiers that perform aerobic heterotrophy; DNRA bacteria would not be present in this zone. A second layer, characterized by alternate oxic and anoxic conditions, would also be thicker than this corresponding zone in environments with high mineralization rates (Fig 5B) and due to the lower C/N ratios, denitrification would likely prevail over DNRA.

In both scenarios, in sediments with low and high mineralization rates, activity in the deeper and strictly anoxic zone where denitrifiers and DNRA can usually grow and be metabolically active, would depend on the balance between electron acceptors (i.e., NO_3_^-^) and organic matter availability [21]. However, in systems with low NO_3_^-^ availability, such as those observed in this study, DNRA bacteria would dominate due to their ability to ferment or utilize other electron acceptors, including sulfate [48]. This proposed model should be considered as a complementary explanation to the dominance or coexistence of DNRA and denitrification processes in sediments.

## Conclusion

Our results show the importance of aerobic organic matter mineralization, which is related to O_2_ consumption and the regulation of substrate availability, to controling N dynamics in aquatic bodies in coastal tropical ecosystems. The presence of microbial mat in the sediment also contributes to regulating O_2_ consumption and N availability due to organic matter assimilation and exudation, thereby influencing N transformations rates. Increases in sediment primary production can lead to a lack of unavailable N for N oxidizers and reducers, as may have occurred at some of our studied sites. Indeed, NO_3_^-^ concentrations were identified as the major regulator of denitrification in the studied environments. This results in general low N transformation rates and gas production, which indicates a low level of N losses via gas emission.
